# Yeast Ist2 Recruits the Endoplasmic Reticulum to the Plasma Membrane and Creates a Ribosome-Free Membrane Microcompartment

**DOI:** 10.1371/journal.pone.0039703

**Published:** 2012-07-09

**Authors:** Wendelin Wolf, Annett Kilic, Bianca Schrul, Holger Lorenz, Blanche Schwappach, Matthias Seedorf

**Affiliations:** 1 Zentrum für Molekulare Biologie der Universität Heidelberg (ZMBH), DKFZ-ZMBH-Allianz, Heidelberg, Germany; 2 Department of Biochemistry I, Universitätsmedizin Göttingen, Göttingen, Germany; 3 Max-Planck-Institut für biophysikalische Chemie, Göttingen, Germany; Simon Fraser University, Canada

## Abstract

The endoplasmic reticulum (ER) forms contacts with the plasma membrane. These contacts are known to function in non-vesicular lipid transport and signaling. Ist2 resides in specific domains of the ER in *Saccharomyces cerevisiae* where it binds phosphoinositide lipids at the cytosolic face of the plasma membrane. Here, we report that Ist2 recruits domains of the yeast ER to the plasma membrane. Ist2 determines the amount of cortical ER present and the distance between the ER and the plasma membrane. Deletion of *IST2* resulted in an increased distance between ER and plasma membrane and allowed access of ribosomes to the space between the two membranes. Cells that overexpress Ist2 showed an association of the nucleus with the plasma membrane. The morphology of the ER and yeast growth were sensitive to the abundance of Ist2. Moreover, Ist2-dependent effects on cytosolic pH and genetic interactions link Ist2 to the activity of the H^+^ pump Pma1 in the plasma membrane during cellular adaptation to the growth phase of the culture. Consistently we found a partial colocalization of Ist2-containing cortical ER and Pma1-containing domains of the plasma membrane. Hence Ist2 may be critically positioned in domains that couple functions of the ER and the plasma membrane.

## Introduction

In *Saccharomyces cerevisiae* large areas of the plasma membrane (PM) are covered with membranes of the endoplasmic reticulum (ER) [1], [2], [3], [4]. In a recent electron tomography study these structures have been described as PM-associated ER [4]. Here, we use the term cortical ER to describe these membranes. The cortical ER consists of interconnected tubules and cisternae, which were found at an average distance of 33 nm from the PM [2], [4].

How the yeast cortical ER is formed remains an open question. The polytopic membrane protein Ist2 is a candidate for a factor involved in this process because it specifically localizes to the cortical ER [5], [6]. Based on sequence homology Ist2 belongs to the TMEM16 or anoctamin (ANO) protein family [7]. Mammalian ANO1 and ANO2 have been identified as Ca^2+^-activated Cl^−^ channels [8], [9], [10]. ANO6 function has been implicated in Ca^2+^-dependent phospholipid scramblase activity [11]. In contrast to yeast Ist2 ANO1, ANO2, and ANO6 reside in the PM [8], [9], [10], [11]. Yeast Ist2 interacts with specific PM lipids, which may contribute to the recruitment of ER to the PM. The interaction with phosphoinositide lipids requires the cortical sorting signal (CSS) at the extreme C terminus of Ist2 and leads to the accumulation of Ist2 in cortical ER domains of yeast and mammalian cells [5], [6], [12]. The induction of cortical ER in mammalian cells by Ist2 expression and localization to cortical ER [12], [13] suggests that Ist2 is an integral part of this membrane. These recent insights argue against the model that Ist2 reaches the PM on a pathway bypassing the Golgi [14], [15].

The PM is organized in domains that function in nutrient transport, signal transduction, and endo- and exocytosis [16], [17], [18], [19], [20]. In *S. cerevisiae* individual PM domains can be visualized by light microscopy. One of these domains, the membrane compartment of Can1 (MCC) contains the H^+^/arginine symporter Can1 and forms dot-like structures, which are 200–300 nm in diameter [21], [22], [23]. In electron microscopy (EM) these structures colocalize with furrow-like membrane invaginations, which are ∼300 nm long [24]. Beside the defining member Can1, the MCC contains the uracil- and tryptophan/H^+^ symporters Fur4 and Tat2, and Nce102, as well as members of the Sur7 protein family [21], [22], [25], [26]. Another domain, the membrane compartment of Pma1 (MCP) contains the H^+^-ATPase Pma1 and forms a large network-like structure [16]. Pma1 pumps H^+^ across the PM and thereby establishes an electrochemical gradient across the yeast PM, which drives the secondary-active uptake of nutrients, e.g. amino acids [27]. Whether and how the segregation of proteins into specific domains of the yeast PM contributes to their function remains unknown.

Here we investigated the function of Ist2 at the interface of ER and PM. We found that Ist2 recruits ER to the PM. This creates a ribosome-free microcompartment between the two membranes. We found evidence for an Ist2-dependent modulation of Pma1 activity under specific conditions. Consistent with this Ist2 partially colocalized with the MCP.

## Results

### The transition from stationary into logarithmic growth phase requires Ist2

**Figure 1:**
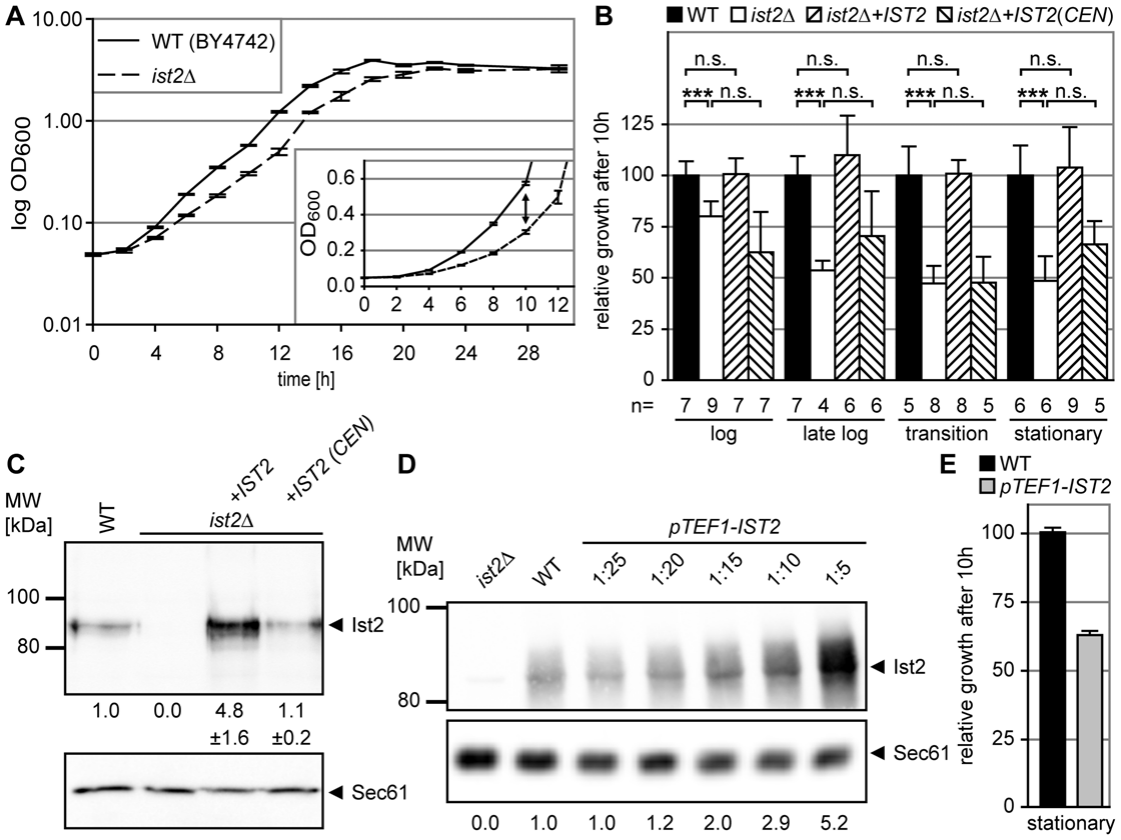
Ist2 is required for the transition from stationary to log phase. (A and B) Growth curves of WT and *ist2Δ* strains in complete synthetic media (HC). The cultures were diluted to 0.05 OD_600_ from stationary cultures (OD_600_>3.0) and incubated at 25°C. Curves in A are semi-log, in B linear showing an enlargement of the initial growth. The arrow indicates the difference between WT and *ist2Δ* after 10 hours. Doubling time for log phase growth was calculated between 0.4 and 0.8 OD_600_. (C) Growth of *ist2*Δ or WT cells transformed with an empty plasmid (pRS303) and of *ist2*Δ with either *IST2* integrated into the *HIS3* locus (*ist2Δ*+*IST2*) or with *IST2* encoded by a *CEN, HIS3* plasmid (*ist2Δ*+*IST2/CEN*). Cultures were diluted to OD_600_ 0.05 from pre-cultures with an OD_600_ 1.0–2.0 (log), OD_600_ 2.0–2.5 (late log), OD_600_ 2.5–3.0 (transition), and OD_600_>3.0 (stationary). Growth relative to WT (set to 100%) after 10 hours is shown. Error bars depict s.d., non-significant differences are indicated as n.s., and triple asterisks show significance (p<0.001). (D) Membranes prepared from 5 OD_600_ of the strains described in C were separated. Ist2 and Sec61 were detected with specific antibodies. The amounts of Ist2 were quantified in membranes from three independent preparations and the s.d. is shown. (E) Relative growth of WT (set to 100%) and *pTEF1-IST2* strains 10 hours after dilution from stationary pre-cultures. Error bars depict s.d.

We characterized the growth of yeast cells lacking the *IST2* gene. This mutant grew similarly to the isogenic wild type strain (WT) and both cultures reached a similar maximal density at stationary growth phase (Fig. 1A). However, the initial growth of *ist2*Δ was impaired during the adaptation from stationary phase to logarithmic (log) phase (Fig. 1A inset, indicated by arrows). During log phase the growth rate of *ist2*Δ accelerated (doubling time 177±33 min) but remained slower than WT (114±5 min).

Comparing the growth of WT and *ist2*Δ, we saw a small but significant growth delay following dilution from a log culture (Fig. 1B, p<0.005). After dilution from late log-, transition-, and stationary phase cultures growth of *ist2*Δ cells was further delayed, when analyzed as growth compared to WT ten hours after dilution (54±10%, 47±14%, 48±15%, p<0.001). Genomic integration of the *IST2* gene into the *his3*Δ*1* locus of *ist2*Δ cells rescued this adaptation defect. Western blot analysis of yeast membranes revealed a 4.8±1.6-fold higher expression of integrated *IST2* as in WT (Fig. 1C). Expression of Ist2 from *CEN* plasmids led to similar expression as in WT (Fig. 1C and Fig. S1B) but did not rescue (Fig. 1B and Fig. S1A). Hence complementation by *IST2* outside the genomic locus requires substantially higher Ist2 protein levels than present in the WT strain. This may be a consequence of *IST2* mRNA transport into the bud [14], [28] and a local function of Ist2.

In order to analyze the phenotype of even stronger Ist2 overexpression we integrated the *IST2* gene under control of the *TEF1* promoter (*pTEF1-IST2*) into the *his3*Δ*1* locus of WT cells. Analysis after serial dilution of membranes from *pTEF1-IST2* revealed 25-fold higher expression as in WT (Fig. 1D). Compared to the isogenic WT the *pTEF1-IST2* strain showed a delayed growth after dilution from a stationary culture (Fig. 1E). As seen for *ist2*Δ the growth of *pTEF1-IST2* accelerated during log phase with a doubling time of 178±13 min (Fig. S1C). Taken together these data suggest that the amount of Ist2 protein is critical for the transition from stationary to log growth.

### Ist2 recruits yeast cortical ER to the PM

Ist2 is a polytopic membrane protein that contains a lipid-binding domain that recognizes PI(4,5)P_2_ and other lipids at the cytosolic face of the PM [5], [6]. Therefore, we hypothesized that Ist2 recruits ER to the PM. The lipid-binding domain is positioned at the C-terminal end of a ∼350 amino acid residues long cytosolic domain, providing enough flexibility to bridge the typical distance of 15 to 50 nm between cortical ER and PM [4].

**Figure 2:**
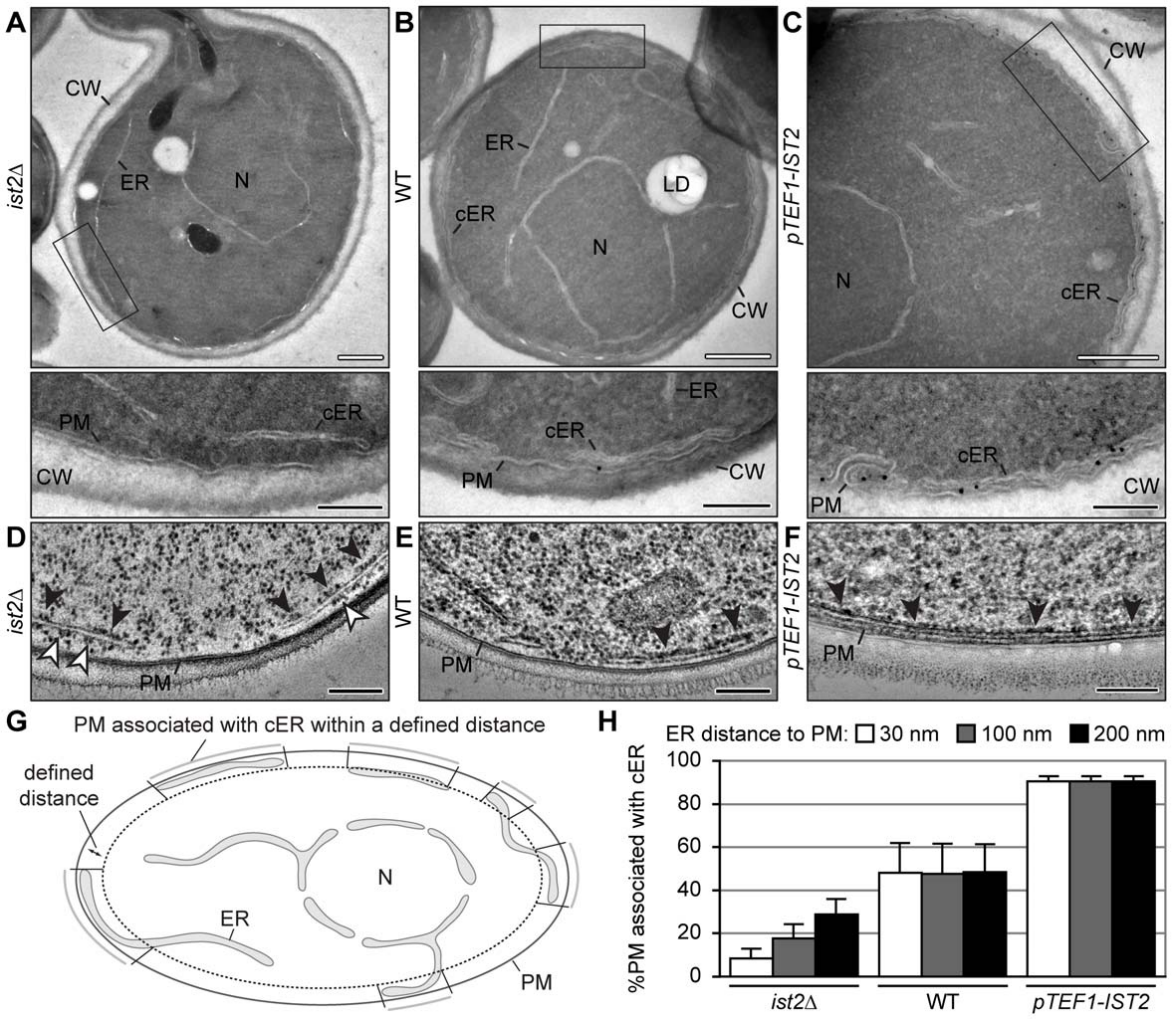
Ist2 recruits yeast ER to the PM. (A–F) Yeast cells expressing different amounts of Ist2 (*ist2Δ* in A and D, WT in B and E, and *pTEF1-IST2* in C and F) were grown to log phase in YPD medium. (A–C) Cells were chemically fixed and ultrathin cryo-sections of gelatine-embedded cells were subjected to immuno-gold labelling using anti-Ist2 antibodies and protein A-10 nm gold. Lower panels show higher magnification of the indicated areas. Please note that cross sections in A–C are shown at different magnification. The average size of *ist2Δ*, WT and *pTEF1-IST2* cells is similar, as revealed by light microscopy (data not shown). (D–F) Cells were high pressure frozen, freeze substituted and Epon embedded. Ribosome-studded ER is indicated by black arrowheads. (D) Ribosomes on the PM face of the cortical ER in *ist2Δ* are indicated by white arrowheads. Organelles and membranes are labelled (PM, plasma membrane; CW, cell wall; ER, endoplasmic reticulum; cER, cortical ER; N, nucleus; LD, lipid droplet). White bars correspond to 500 nm and black bars to 200 nm. (G) Cartoon illustrating the morphology of ER. For quantification PM and ER were traced in electron micrographs of representative cells after chemical fixation. The relative amount of PM associated with cortical ER within a defined distance (dotted line) was analyzed. (H) Quantification of PM fraction associated with cortical ER within a distance of 30 nm (white), 100 nm (grey), and 200 nm (black) in the indicated strains. Values represent the mean of independent images (*ist2Δ:* n = 12; WT: n = 10; *pTEF1-IST2*: n = 9). Error bars indicate the s.d.

To test our hypothesis, we performed immuno-EM with chemically fixed cells and detected the localization of Ist2. In all cells we observed perinuclear ER structures surrounding the nucleus (Fig. 2, labeled as N). In addition, we saw ER tubules located more to the centre of the cell (ER) and cortical ER (cER), which is positioned close to the PM and the cell wall (CW). Immuno-staining of WT cells with 10 nm gold-labeled Ist2-specific antibodies occasionally led to detection of Ist2 at sites where ER and PM were opposed (Fig. 2B, inset). In *pTEF1-IST2* cells, we observed a strong accumulation of gold particles at the cortical ER and the PM (Fig. 2C, inset), suggesting that Ist2 localizes to cortical ER associated with the PM. The deletion of *IST2* led to an increased distance between ER and PM (Fig. 2A, D).

To quantify the amount of cortical ER in the three different strains, we defined ER membranes in proximity to the PM within distances of either 30, 100 or 200 nm. For each cell, we measured the total PM length in a section as well as the length of PM with underlying cortical ER within the defined threshold as schematically illustrated in Fig. 2G.

Finally, we calculated the percentage of PM that associates with cortical ER in each cell. This quantification revealed that in WT cells 48% of the PM is covered with cortical ER within 30 nm distance from the PM. Overexpression of Ist2 led to increased association of ER with the PM such as 91% of the PM had an underlying cortical ER (Fig. 2H). Indeed, the cortical ER covered most parts of the PM except invaginations (Fig. 2C, inset). In both cases theses values did not differ after quantifying ER membranes within 100 or 200 nm distance from the PM demonstrating that cortical ER associated within a distance of 30 nm with the PM.

In *ist2*Δ cells the amount of PM-associated cortical ER is substantially reduced. Compared to 48% in WT cells only 8% of PM is covered with cortical ER when quantified within a distance of 30 nm (Fig. 2H). Quantifying ER membranes within increasing distances to the PM from 30 nm to 100 nm and 200 nm revealed that in *ist2*Δ some cortical ER can be found at larger distances from the PM. In comparison to WT and *pTEF1-IST2* cells in which the amount of cortical ER stays constant within all defined distances, this increase demonstrates that cortical ER is less close associated with the PM in *ist2*Δ cells.

As an independent method for the quantification of PM-associated ER, we employed fluorescence light microscopy and measured the length of the PM with cortical ER staining in cross sections. For this purpose, we expressed three types of GFP-tagged ER proteins: a soluble luminal protein (GFP-HDEL), an integral membrane protein (Sec63-GFP), and a tail-anchored protein (GFP-Ubc6). We found that overexpression of Ist2 in *pTEF1-IST2* cells led to an increased amount of PM with associated cortical ER (Fig. S2A), in agreement with the EM data. In *ist2*Δ and WT cells, however, we observed no difference in the association of the cortical ER with the PM. This is consistent with previous results [6], showing that deletion of *IST2* did not lead to a reduction of cortical ER staining within the resolution of standard light microscopy. This difference could be explained by the accumulation of peripheral ER at a distance to the PM that is not resolved by light microscopy, where the maximal resolution limit is 200 nm. Indeed, using 200 nm as a threshold to quantify cortical ER in our EM pictures revealed that 48.5% (±9.7%) of the WT PM and as much as 29% (±6%) of the *ist2*Δ PM is covered with cortical ER. The remaining discrepancy between values obtained by light microscopy and EM results either from the fact that the resolution limit in our light microscopy is larger than 200 nm or from the technical difference of using fixed cells for EM and living cells for light microscopy. In live-cell imaging by light microscopy additional fluorescence from extra-focal planes is recorded. This clearly demonstrates that the resolution achieved by EM is critical to determine the amount of Ist2-dependent cortical ER.

To better preserve the ultra structure of the yeast cells and to analyze ER morphology, *ist2*Δ, WT and *pTEF1-IST2* cells were vitrified using high pressure freezing, freeze substituted and Epon embedded. Analyses of membranes in such cells showed that the cortical ER is studded with ribosomes, which were seen as black dots (Fig. 2D–F, indicated by arrowheads). In WT and *pTEF1-IST2* cells the ribosomes were restricted to the cytosolic face of the cortical ER (Fig. 2E, F). The space between ER and PM was free of ribosomes and appeared transparent. In *ist2*Δ this was seen at some parts of the cortical ER but most of the cortical ER was covered with ribosomes on both sides (Fig. 2D, indicated by white arrowheads).

The loss of Ist2 led to an increased distance between ER and PM, which allows ribosomes with a diameter of approximately 25 nm to occupy the space between ER and PM. On the other hand the overexpression of Ist2 resulted in an increase of cortical ER tightly opposed to the PM. Taken together, these data support a function of Ist2 as a membrane tether or as a protein that enables tethering performed by other components. Ist2-mediated recruitment of ER to the PM may create a specific ribosome-free subcompartment of the cytosol positioned between two adjacent membranes.

### Increased ER-PM contact formation by overexpression of Ist2 traps the nucleus at the PM

**Figure 3:**
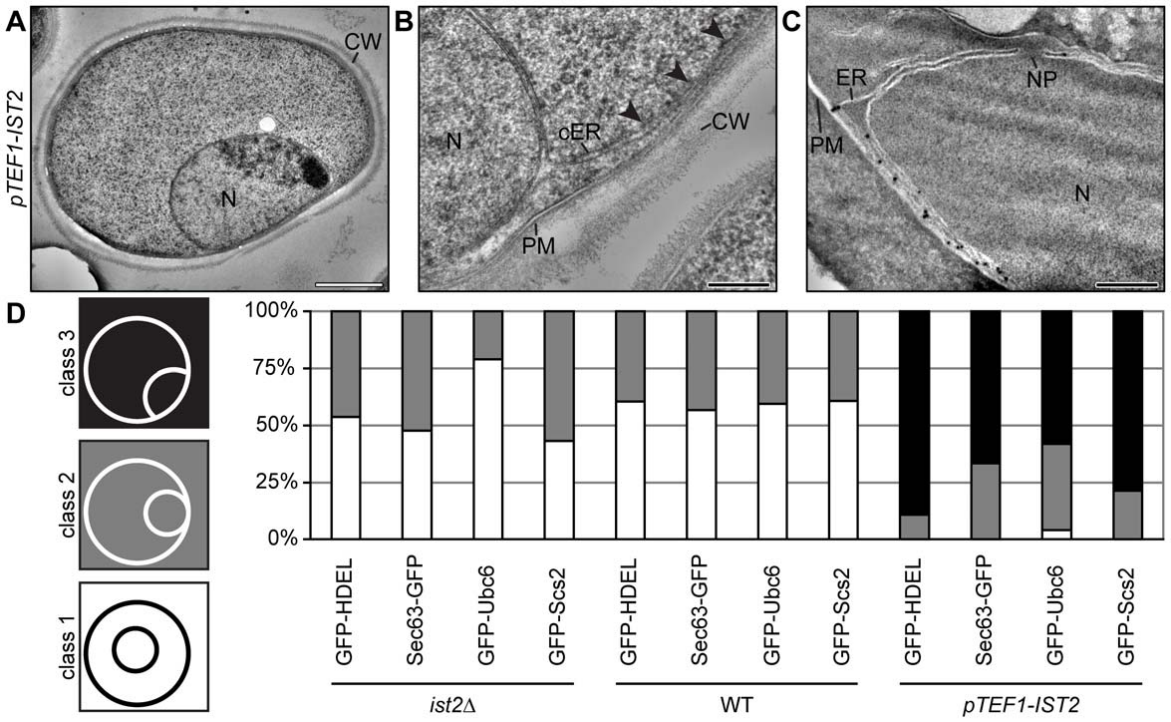
Overexpression of Ist2 traps the perinuclear ER at the PM. (A–C) Electron micrographs of log phase *pTEF1-IST2* cells. (A and B) Cells were high pressure frozen, freeze substituted and Epon embedded. (B) Arrowheads indicate the association of cortical ER with the PM. (C) Immuno-gold labeling of chemically fixed cells using anti-Ist2 antibodies. Organelles and membranes are labeled (PM, plasma membrane; CW, cell wall; ER, endoplasmic reticulum; N, nucleus; NP, nuclear pore). The white bar corresponds to 1 µm and the black bar to 200 nm. (D) Association of nuclei analyzed by CLS microscopy of *ist2Δ*, WT, and *pTEF1-IST2* cells expressing GFP-HDEL (n = 39, 38, 55), Sec63-GFP (n = 46, 60, 48), GFP-Ubc6 (n = 66, 54, 50), and GFP-Scs2 (n = 52, 74, 95). Class 1 cells have the nucleus in the centre without any contact to the periphery (white), class 2 cells have the nucleus in contact with the periphery (grey), and class 3 cells have a deformed nucleus that aligns with the periphery (black). Quantification was performed with blinded data.

In cells overexpressing Ist2, we often observed an association of the nuclei with the cell periphery (Fig. 3A–C). The nuclear envelope was associated with the PM over a distance of more than 1 µm. This tight association of perinuclear ER and PM resulted in deformation of the nucleus. Cortical ER that emanated from the perinuclear ER became associated with the PM (Fig. 3B, indicated by arrowheads). Immuno-gold labeling with Ist2 antibodies showed staining at regions where the nuclear envelope aligns with the PM but not at other regions of the nuclear envelope. This suggests that Ist2 specifically localizes to ER domains that are in close proximity to the PM (Fig. 3C).

In order to quantify this effect of Ist2 overexpression, we analyzed the nuclear morphology in *ist2*Δ, WT and *pTEF1-IST2* cells expressing GFP-HDEL, Sec63-GFP, GFP-Ubc6, or GFP-Scs2 by confocal laser scanning (CLS) microscopy. Cells were divided in three classes with (1) the nucleus in the centre without any contact to the periphery, (2) the nucleus in contact with the periphery, and (3) a deformed nucleus in contact with the periphery (Fig. 3D). Examples for the localization of the individual ER proteins in all classes are shown in figure S2C. Overexpression of Ist2 caused the accumulation of deformed nuclei at the cell periphery in the majority of cells (class 3), and nuclei of almost all remaining cells also showed contacts to the periphery (class 2). In contrast, fewer contacts between the nuclei and the periphery were detected in WT and *ist2*Δ cells.

Next, we asked whether Ist2 contributes to the inheritance of ER into daughter cells and coexpressed Sec63-GFP and Yop1-mCherry in *ist2*Δ, WT and *pTEF1-IST2* cells. Sec63 is an integral membrane protein involved in posttranslational protein translocation. Yop1 is a member of the reticulon protein family and insertion of its hairpin-like hydrophobic region into the cytosolic face of ER induces membrane curvature [29]. The expression of Yop1-GFP was decreased in *ist2*Δ but the protein remained peripherally localized (Fig. S3E and F). Overexpression of Ist2 led to accumulation of Yop1-GFP in large cortical structures resembling cisternae but did not change the expression level. A comparison of the fluorescence ratios between daughter and mother cells revealed an accumulation of Yop1-mCherry in small daughter cells, whereas Sec63-GFP was equally distributed (Fig. S3A and B). This indicates a sequential inheritance of specific ER domains into daughter cells [4]. The deletion of *IST2* had no effect, whereas overexpression of Ist2 had only a small effect on the inheritance of ER into small daughters (Fig. S3A). As described above, overexpression of Ist2 caused an accumulation of Sec63-containing perinuclear ER at the cell periphery (Fig. S3D). In summary, these data suggest that proper ER morphology and position of the nucleus are sensitive to the abundance of Ist2 whereas inheritance of ER into daughter cells is not affected.

### Partial overlap of Ist2 with membrane proteins of the cortical ER

**Figure 4:**
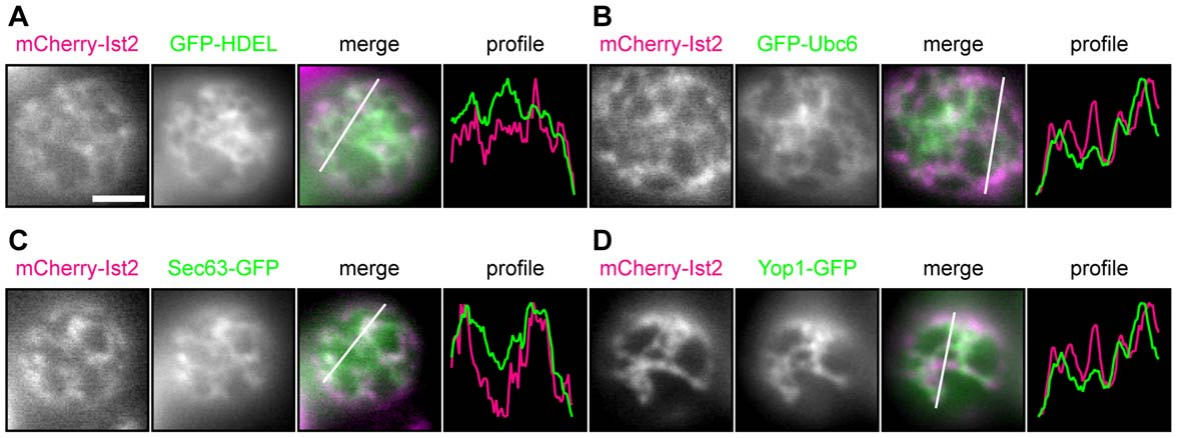
Ist2 colocalizes with proteins of the cortical ER. Surface views of epifluorescence microscopy with *ist2Δ* cells expressing mCherry-Ist2. These cells co-express GFP-HDEL, Sec63-GFP, GFP-Ubc6 from *CEN* plasmids or Yop1-GFP. The merged images show mCherry-Ist2 in magenta and the respective ER proteins in green. The fourth panels show intensity profiles of the indicated cross sections in the merged images.

Since Ist2 is involved in the recruitment of ER to the PM, we investigated its localization within the cortical ER and compared it to the localization of other ER proteins. Variations in the distance between cortical ER and PM have been reported [4]. Cortical ER domains with Ist2 may represent specific sites in close contact to the PM. Genomic integration of GFP-Ist2 or mCherry-Ist2 in *ist2*Δ rescued the delayed transition from stationary into log phase, indicating that GFP- and mCherry-Ist2 proteins are functional (Fig. S4A). The surface views of mCherry-Ist2 showed localization in interconnected patch-like structures (Fig. 4). This network surrounded distinct areas free of Ist2 and other ER proteins. Such localization is typical for proteins in the cortical ER [30]. The mCherry-Ist2 signals (magenta in merged images in Fig. 4) partially overlapped with the artificial luminal ER marker GFP-HDEL, with the tail-anchored protein GFP-Ubc6, with Sec63-GFP, and with Yop1-GFP (green in merged images). The Pearson's coefficient for colocalization ranged from 0.616±0.075 to 0.728±0.069 (Fig. S4B). Colocalization of dsRed-HDEL with Sec63-GFP and GFP-Ubc6 revealed a better overlap (Pearson's coefficient of 0.949±0.035 and 0.95±0.037; Fig. S4B, E and G), suggesting a partial segregation of Ist2 into subdomains of the cortical ER. However, the segregation of membrane proteins in the cortical ER was not specific for Ist2. The colocalization of Yop1-mCherry with GFP-HDEL was similar to mCherrry-Ist2 with GFP-HDEL (Pearson's coefficient of 0.786±0.07 and 0.616±0.075; Fig. S4B, C and D). Colocalization of Sec63-GFP and GFP-Ubc6 with Yop1-mCherry showed Pearson's coefficients of 0.716±0.125 and 0.797±0.084 (Fig. S4B, F and H).

### Association of Ist2 with domains of the PM

**Figure 5:**
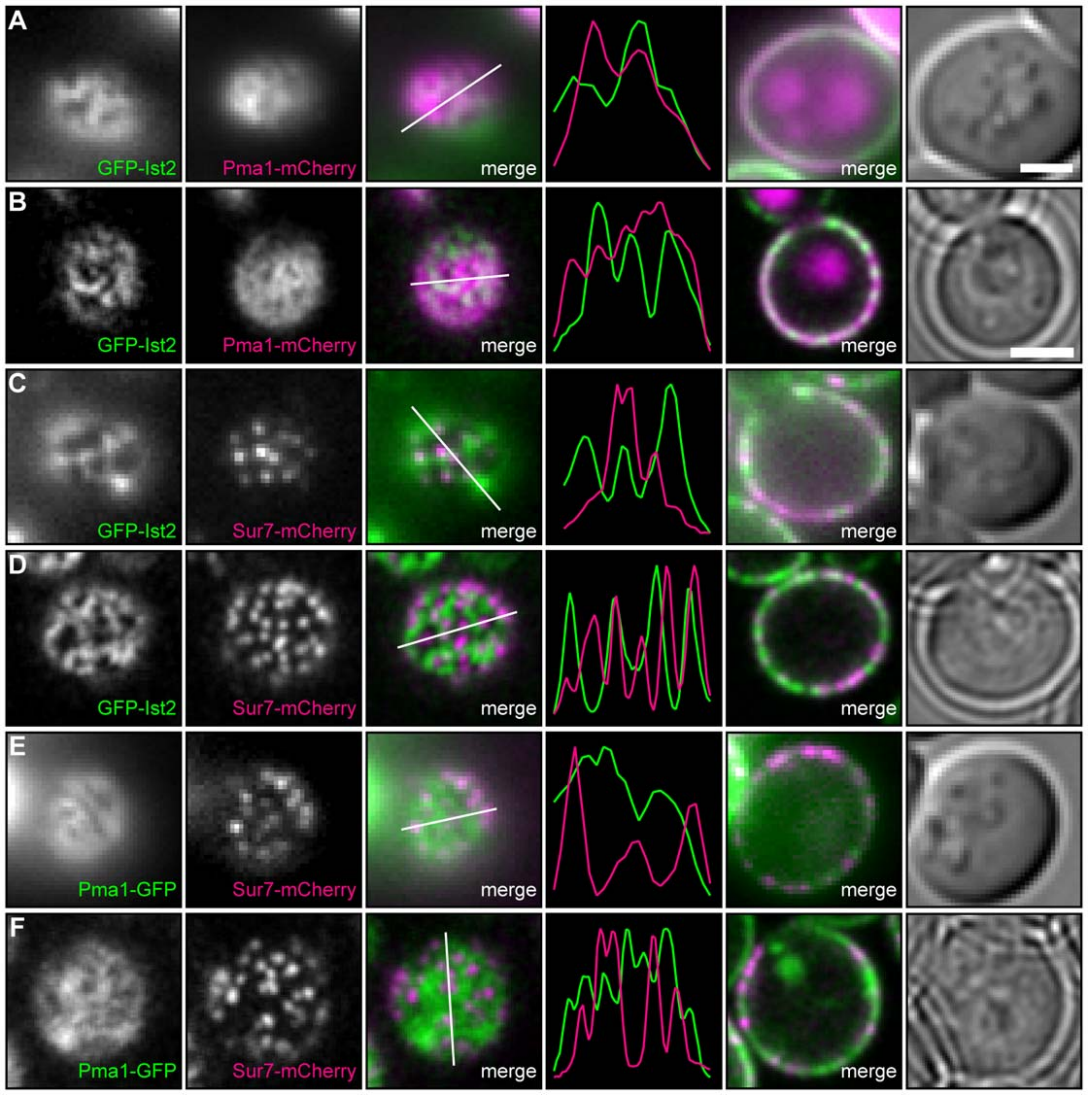
Partial colocalization of Ist2 and Pma1 in network-like structures. Cells were grown in complete HC media and imaged by TIRF (A, C and E) and CLS (B, D and F) microscopy. The third panel shows the merge image of the first and second panel with an intensity profile of the indicated cross section in the fourth panel. (A, C and E) Merged view of equatorial sections in epifluorescence mode (panel 5) and corresponding DIC images of the TIRF images (panel 6). (B, D and F) Merged view of equatorial sections and phase contrast images are shown in the fifth and sixth panels. (A and B) The *PMA1* gene was replaced by *PMA1-mCherry* in *ist2Δ* cells with *GFP-IST2* integrated into the *his3Δ1* locus. The localization of GFP-Ist2 (green in the merged image) and Pma1-mCherry (magenta in the merged image) was analyzed. (C and D) The *SUR7* gene was replaced by *SUR7-mCherry* in *ist2Δ* cells with *GFP-IST2* integrated into the *his3Δ1* locus. The localization of GFP-Ist2 (green in the merged image) and Sur7-mCherry (magenta in the merged image) was analyzed. (E and F) *PMA1* and *SUR7* were replaced by *PMA1-GFP* and *SUR7-mCherry* in WT cells and the localization of Pma1-GFP (green in the merged image) and Sur7-mCherry (magenta in the merged image) was analyzed.

The impaired adaptation of *ist2Δ* cells, when diluted from cultures with an OD_600_>2.0 (Fig. 1A–C) may result from a functional coupling of Ist2 with proteins in specific PM domains. The yeast PM consists of two main compartments, namely the MCP and the MCC [16], [22], [25]. To test whether Ist2 colocalizes with proteins of the MCP we employed total internal reflection fluorescence (TIRF) and CLS microscopy. We replaced the *PMA1* gene with *PMA1-mCherry* in *ist2*Δ cells with *GFP-IST2* integrated into the *his3*Δ*1* locus. TIRF microscopy and a surface view of CLS microscopy confirmed the network-like localization of GFP-Ist2 (Fig. 5A–D). The merged signals (white) showed a partial overlap of GFP-Ist2 (green) with Pma1-mCherry (magenta) (Fig. 5A, B, merge). A Pearson's coefficient of 0.896 (n = 19) in TIRF microscopy is consistent with partial colocalization of Ist2 and Pma1. Equatorial views (Fig. 5A, B, fifth panel) also revealed a partial overlap of both signals in the periphery while internalized Pma1-mCherry localized to vacuoles. However, certain areas containing GFP-Ist2 were clearly separated from Pma1-mCherry. Partial colocalization of Ist2 and Pma1 was also illustrated by intensity profiles of GFP-Ist2 and Pma1-mCherry signals (Fig. 5A, B, fourth panel, indicated by white lines in third panel).

**Figure 6:**
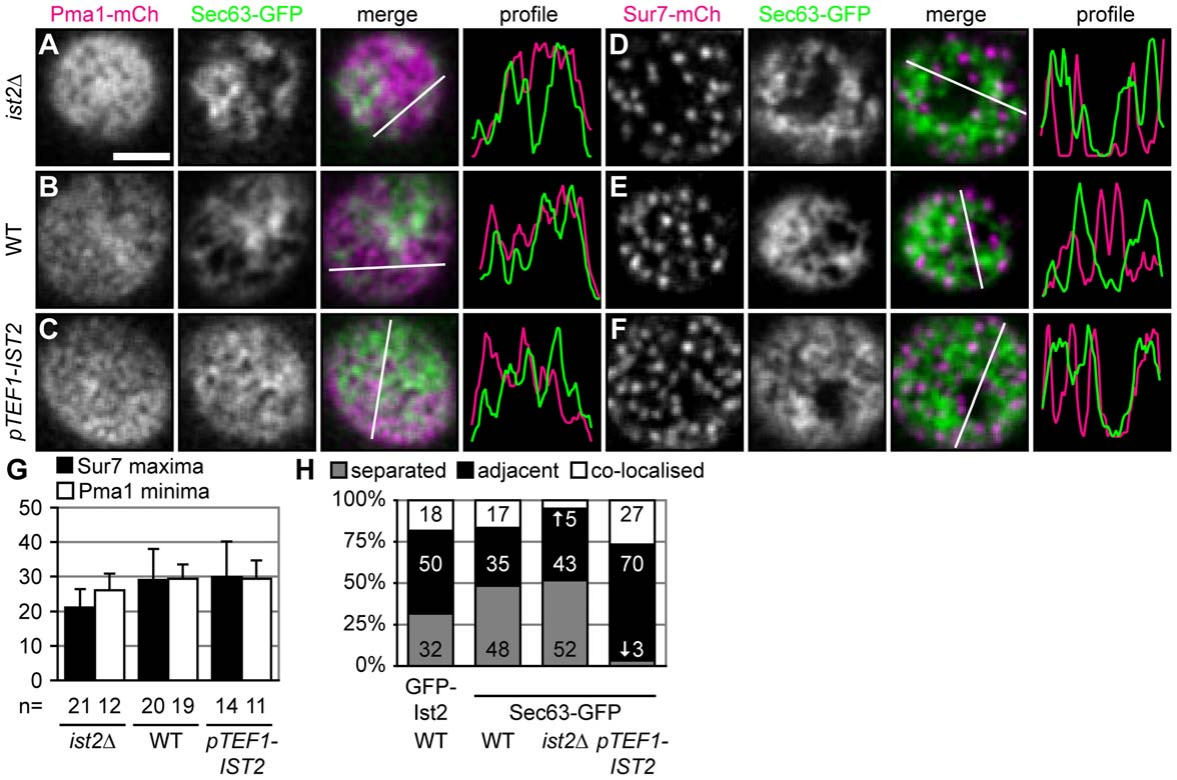
Ist2 dependent proliferation of the cortical ER does not alter PM compartmentation. (A–F) Surface views taken by epifluorescence microscopy showing *ist2Δ*, WT and *pTEF1-IST2* cells coexpressing Pma1-mCherry (A–C) or Sur7-mCherry (D–F) and Sec63-GFP. The merged images show Pma1-mCherry and Sur7-mCherry in magenta and Sec63-GFP in green. The fourth panels show intensity profiles of the indicated cross sections in the merged images. (G) Mean number of Sur7-mCherry maxima (black) and Pma1-mCherry minima (white) in *ist2Δ*, WT and *pTEF1-IST2* cells. Error bars depict s.d.. (H) Quantification of single Sur7-mCherry domains separated from (grey), adjacent to (black), and colocalized with cortical ER (white). Corresponding GFP-labelled ER proteins and strains are indicated.

We used Sur7-mCherry in order to test whether the GFP-Ist2 network overlaps with dot-like MCC structures. In TIRF microscopy Sur7-mCherry localized in dots with a size of 1 or 2×2 pixels (Fig. 5C). With the given resolution of the camera, where pixel sizes correspond to 160 nm, this fits well to the reported dimension of a Sur7 domain when analyzed by light microscopy [21], [22]. A similar distribution of Sur7-mCherry was observed in surface views of CLS microscopy (Fig. 5D and Fig. S5A–D). Consistent with previous reports the dot-like Sur7-mCherry domains were segregated from the Pma1-GFP network (Fig. 5E, F). Most of the Sur7-mCherry domains were adjacent to or separated from the network-like GFP-Ist2 structures (Fig. 5C, D, merged images and intensity profiles). Since GFP-Ist2 signal covered 52% of the cell surface (Fig. S5E), one would expect that half of the Sur7 domains overlap with Ist2 if the distribution is random. However, only 18% of single Sur7-mCherry domains were colocalized (Fig. 6H), suggesting a non-random distribution. This is consistent with a random distribution of MCC in areas devoid of cortical ER [20].

Next, we analyzed if the proportion of the ER that colocalized with Pma1 and Sur7 changes in *ist2*Δ and *pTEF1-IST2* cells. As expected in cells overexpressing Ist2, we observed a proliferation of Sec63-GFP containing cortical ER (Fig. 6C and F). In these cells the cortical ER covered 79% of the cell surface, compared to 37 and 59% in *ist2*Δ and WT cells, respectively (Fig. S5E). Changes in Ist2 expression had only minor effects on the number of MCC domains (Fig. 6G). In WT and *ist2Δ* most of the Sur7-mCherry domains were adjacent to or separated from the network-like Sec63-GFP structures, as previously seen for Ist2 in WT (Fig. 6D–F, H). In *ist2*Δ the colocalization of Sur7-mCherry and Sec63-GFP decreased, whereas it increased in *pTEF1-IST2* (Fig. 6H). Moreover, overexpression of Ist2 led to reduced segregation of Sur7-mCherry and Sec63-GFP. In *ist2Δ* and *pTEF1-IST2* strains the number of colocalized Sur7-mCherry domains was smaller than expected from the surface area covered with Sec63-GFP (Fig. 6H and S5E). This suggests that the distribution of Sur7 domains and cortical ER is not random. Most of the Sur7 domains are separated from or adjacent to the cortical ER and this specific distribution does not require Ist2.

### Ist2 is required for rapid glucose-induced Pma1 activation

If Ist2 functions at ER-PM contact sites, it is possible that such function influences metabolic processes at the PM. Pma1-mediated pumping of H^+^ across the PM is such a candidate process. It controls the cytosolic pH and is involved in the acidification of the growth medium. The activity of Pma1 consumes at least 20% of the cellular ATP and is subject to strict regulation by extracellular glucose [31], [32], [33].

**Figure 7:**
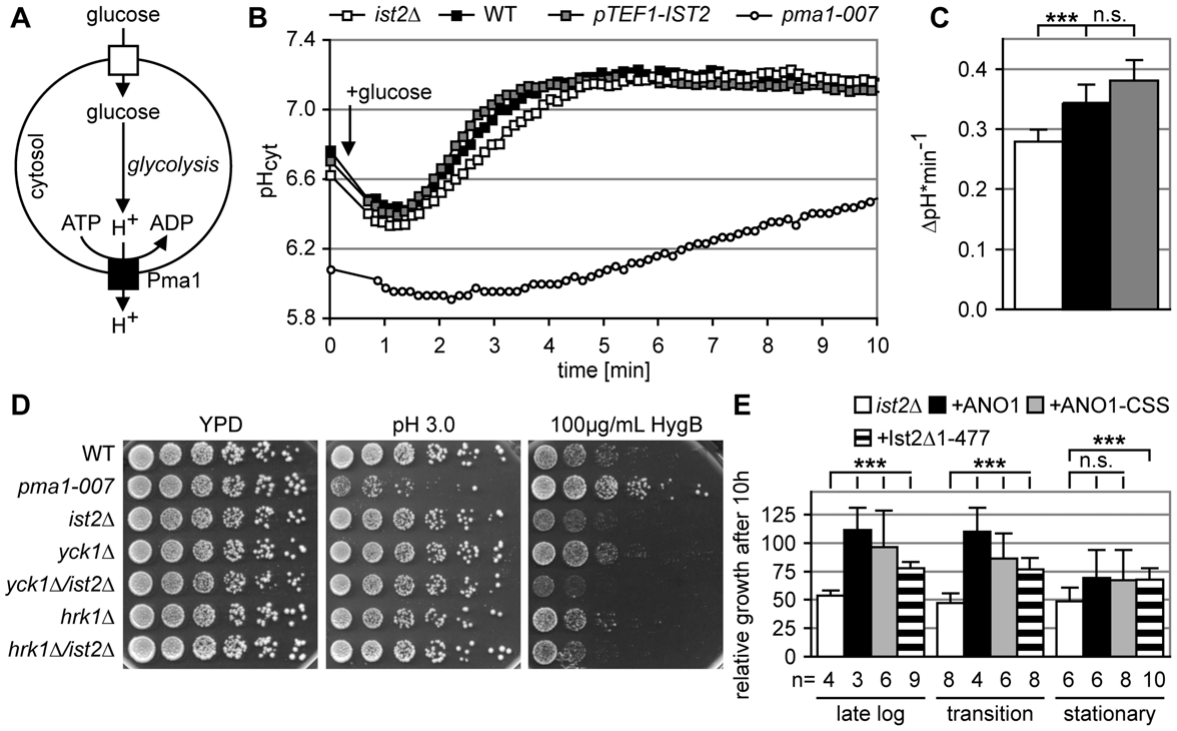
Ist2 contributes to the regulation of Pma1 activity. (A) Cartoon illustrating glucose-induced H^+^ pumping by Pma1. (B) Kinetic measurement of cytosolic pH in *ist2*Δ, WT, *pTEF1*-*IST2,* and *pma1-007* strains. Cells were starved for glucose for 1 hour in synthetic media. At the indicated time, marked with an arrow, 2% glucose was added. (C) Quantification of the rate of H^+^ pumping shown as ΔpH/min. (D) Five-fold serial dilutions of the indicated strains on plates with YPD, YPD adjusted to pH of 3.0, and YPD+100 µg/ml hygromycin B. (E) Growth of *ist2Δ* and *ist2Δ* expressing ANO1, ANO1+CSS or mCherry-Ist2Δ1–477 during the first 10 hours after dilution from pre-cultures with an OD_600_ of 2.0–2.5 (late log), 2.5–3.0 (transition), and >3.0 (stationary). Growth of the corresponding WT was set to 100%. In (C) and (E) error bars depict s.d., non-significant differences are indicated as n.s., and triple asterisks show significance (p<0.001).

In order to analyze the effect of Ist2 on Pma1 activity, we measured the alkalinization of the cytosol in response to Pma1 activation by glucose (Fig. 7A). Dynamic changes of the cytosolic pH in living cells expressing the pH-sensitive GFP derivate ratiometric pHluorin were measured in a plate reader [34]. In response to glucose starvation the cytosolic pH (pH_c_) decreased to 6.77 and 6.71 in WT and *pTEF1-IST2* (Fig. 7B). This pH_c_ decrease was more pronounced in *ist2*Δ (pH 6.63). The pH_c_ of the *PMA1* hypomorph *pma1-007* was 6.08, consistent with a reduced amount of Pma1 in these cells [35]. Except for *pma1-007* addition of glucose (Fig. 7B, indicated with an arrow) resulted in rapid decrease of pH_c_, most likely, a consequence of H^+^ production by glycolysis (see cartoon Fig. 7A). About one min after glucose addition the cytosol was alkalinized to a constant pH_c_ of 7.1–7.2 within five min (Fig. 7B). Alkalinization depends on glucose-mediated activation of Pma1. Addition of galactose to glucose-starved cells did not stimulate Pma1 activity (Fig. S6A). Importantly, the amount of Ist2 expression influenced this rate of pH_c_ change. Deletion of *IST2* led to significantly slower alkalinization of the cytosol as compared to WT (p<0.05), whereas overexpression of *IST2* resulted in a slightly but not significantly faster alkalinization (quantification in Fig. 7C). The amount of Pma1 did not change significantly in cells with different amounts of Ist2, whereas *pma1-007* showed decreased Pma1 expression (Fig. S6B, and [35]). Thus, Ist2 influences H^+^ pumping in response to glucose.

The activity of Pma1 is under strict control. Growth at acidic pH requires strong activation of Pma1 [35]. Cells with reduced Pma1 expression display a growth defect at pH 3.0, as seen for *pma1-007* (Fig. 7D). Down-regulation of Pma1 activity also results in a reduced uptake of toxic cations [36], [37]. Consequently *pma1-007* grows better than WT in the presence of a toxic concentration of the cation hygromycin B [38], [39]. Deletion of *IST2* led to impaired growth in media containing hygromycin B, but had no effect on growth at pH 3.0 (Fig. 7D) indicating that Pma1 function is intact. In order to further test whether Ist2 function is required under growth conditions where Pma1 activity is regulated, we combined *ist2*Δ with the deletion of *YCK1* and tested growth on hygromycin B and at low pH. Yck1 is a PM-bound casein kinase I that down-regulates Pma1 activity in response to glucose starvation [40], [41]. The double mutant showed an enhanced growth defect on hygromycin B, whereas growth on YPD and on YPD pH 3.0 was similar to WT (Fig. 7D). This synthetic growth defect was specific, as the deletion of the positive Pma1 regulator Hrk1 kinase [42] had no effect (Fig. 7D). The observed negative genetic interaction between *IST2* and *YCK1* is consistent with the hypothesis that presence of Ist2 influences Pma1 activity.

Another study reported a negative genetic interaction between *IST2* and *BTN1* or *BTN2* double mutants at high NaCl concentration [43]. Btn1 is the yeast orthologue of human CLN3, which is involved in neuronal ceroid lipofuscinosis characterized by an accumulation of storage material in lysosomes [44]. Btn2, which is up-regulated in *BTN1* mutants, functions as a SNARE and retromer-interacting protein that is involved in the recycling of cargo proteins from late endosomes to the Golgi [45]. In order to clarify this aspect of Ist2 function, we analyzed the growth of *ist2*Δ, *btn1*Δ and *btn2*Δ single and double mutants at 1 M NaCl concentration. Only the single *btn2*Δ mutant showed a slight growth defect on YPD plates with 1 M NaCl, whereas all other mutants grew at least as well as the WT (Fig. S6C). This was independent of *LYS2* and *MET15* genotypes in BY4741 and BY4742 strains. Our results contradict the previously reported growth defect of *ist2Δ/btn1Δ* and *ist2Δ/btn2Δ* double mutants on high salt [43].

The putative mammalian Ist2 orthologues ANO1 and ANO6 function as a Ca^2+^-activated Cl^−^ channel or are involved in phospholipid scramblase activity, respectively [8], [9], [10], [11]. Therefore, we tested whether changes in Ca^2+^ homeostasis or signaling had an influence on the growth of *IST2* mutants. The Ca^2+^ ATPase Pmr1 pumps Ca^2+^ from the cytosol into ER and Golgi and its deletion results in increased cytosolic Ca^2+^ concentration [46]. Double deletion of *PMR1* and *IST2* led to a salt-dependent growth defect at acidic pH (Fig. S6D). In order to interfere with Ca^2+^ signaling, we deleted *CNB1*, the regulatory subunit of the Ca^2+^/calmodulin-regulated type 2B protein phosphatase calcineurin [47]. This deletion resulted in a salt- and alkaline pH-dependent growth defect but additional deletion of *IST2* did not change this phenotype (Fig. S6D). Hence by employing growth assays we were not able to uncover interplay between *IST2* function and Ca^2+^ signaling.

Finally, we tested whether the expression of human ANO1 under control of the *IST2* untranslated regions rescues the delayed growth observed for *ist2*Δ after dilution from pre-cultures with OD_600_>2.0. Although we failed to detect the expression of ANO1 in yeast by western blotting using ANO1-specific antibody (Fig. S6E), we could observe a partial rescue (Fig. 7E). An activity at very low expression levels would be consistent with the function of Ist2 and ANO1 as ion channels. A few copies of these proteins would be able to affect the ion composition of a small volume such as the membrane-enclosed cytosol between the PM and the cortical ER. When *ist2*Δ cells were grown to an OD_600_ of <3.0 (Fig. 7E, late log and transition), the integration of ANO1 rescued the delayed adaptation to log phase growth. These cells reached 111±20% and 110±21% of the WT growth, respectively (Fig. 7E, p<0.05). Under these conditions *ist2*Δ reached 50±2% and 44±7%, respectively. After dilution from stationary pre-cultures with an OD_600_ of >3.0 this rescue was not observed. The addition of the CSS from Ist2 to ANO1 did not improve its ability to rescue (Fig. 7E). Expression of a N-terminally mCherry-tagged, truncated version of Ist2, lacking the N-terminal domain and TMDs 1–6 (amino acids 1–477 are deleted; mCherry-Ist2Δ1–477 in Fig. 7E), in the cortical ER (Fig. S6F) partially rescued the *ist2*Δ phenotype (Fig. 7E). This suggests that the Ist2 function comprises both recruitment of ER to the PM and a function as ion channel.

Taken together, our findings suggest a role of Ist2 in the recruitment of ER to the PM and in the functional coupling of ion transport processes between these membranes.

## Discussion

We found that the membrane protein Ist2 recruits ER to the PM. Ist2 activity in the cortical ER modulates glucose-induced H^+^-pumping across the PM. Since Ist2 is required for rapid growth after dilution from pre-cultures with an OD_600_>2.0, Ist2-mediated coupling of ER and PM may be involved in the adaptation of cells to changing growth conditions.

Ist2 is an integral membrane protein, the vast majority of which localizes to the cortical ER [5], [6]. Localization of Ist2 to specific cortical ER domains is supported by a strong but not complete overlap with other ER proteins at the cell periphery. Yop1 is concentrated at edges of cortical ER in regions with high membrane curvature [30]. Like Ist2 it showed only partial overlap with other ER proteins. However, Ist2 and Yop1 did not localize to identical domains, suggesting an accumulation in distinct subdomains of the cortical ER. As an integral component of the ER membrane, Ist2 tightly connects peripheral ER structures with the PM. This may be achieved by direct binding of Ist2 to phosphoinositide lipids at the cytosolic face of the PM or by the recruitment of other factors that are involved in the association of ER and PM. The abundance of Ist2 determines the amount of cortical ER that is associated with the PM and sets the distance between cortical ER and the PM. Ist2 may function locally, since its mRNA is transported as ribonuclear particle to the bud tip and expression in the bud depends on mRNA transport [14], [28]. This may explain why the function of Ist2 depends critically on the abundance, when Ist2 is expressed outside the native genomic locus. Since cortical ER structures less closely associated with the PM remain in *ist2Δ* cells, factors other than Ist2 must contribute to the formation of cortical ER. Strong overexpression of Ist2 resulted in the proliferation of cortical ER covering the PM almost completely. This overexpression of *IST2* mRNA may saturate mRNA transport and lead to accumulation of Ist2 at the perinuclear ER and to alignment of the nucleus with the PM. Deletion of Ist2 did not result in defects of ER inheritance into daughter cells. However, binding of newly synthesized Ist2 to PM lipids may transform inherited ER in the daughter cell into cortical ER that is closely associated with the PM. Such conversion of ribosome-covered ER into cortical ER, with ribosomes restricted to the cytosolic face, is supported by recent electron tomography experiments [4].

Deletion of *IST2* reduced the amount of cortical ER and increased the distance between ER and PM, so that ribosomes gain access to the space between ER and PM. In WT the space between cortical ER and PM lacks ribosomes. A similar ribosome-free space has been described for thin cortical ER in HeLa cells expressing STIM1 protein [48]. Therefore, this space may represent a specific microenvironment with a yet unknown protein and ion composition that is functionally separated from the surrounding cytosol [4]. Ion depletion effects in a small membrane-enclosed space have been discussed and investigated for the synaptic cleft found in the mammalian nervous system. Indeed, extensive activation of neurotransmitter-receptors in the postsynaptic membrane can deplete extracellular calcium ions in the cleft [49]. This is of conceptual interest because (depending on the synapse studied) the dimensions of the cleft between a pre- and a postsynaptic membrane can be comparable to the dimensions of the Ist2-dependent space between cortical ER and PM described here.

Consistent with the idea of a functional coupling between ER and PM in tightly apposed domains we found that loss of *IST2* led to increased acidification of the cytosol under glucose-starvation and to slower alkalinization in response to glucose stimulation. A function of Ist2 as an ion channel could influence cytosolic pH (pH_c_), which may control pH-sensitive kinases modulating Pma1 activity [41]. Ist2-dependent association of ER with the PM may contribute either directly or indirectly to glucose-induced activation of Pma1 in glucose-starved cells. A regulatory role of Ist2 in the control of Pma1 activity is supported by a negative genetic interaction between *ist2Δ* and *yck1Δ* double mutants under conditions that require low Pma1 activity. Yck1 is one of two PM-bound casein kinases I in yeast that phosphorylate and down-regulate Pma1 under glucose starvation [41]. Whereas the measurement of pH_c_ revealed a stimulatory function of Ist2 on Pma1 activity, the genetic analysis suggested an inhibitory function. These contradictory findings may be explained by differences in Pma1 activation by glucose [33]. In contrast to the measurements of pH_c_, where glucose-starved cells were exposed to glucose, the growth of double mutants was analyzed in constant presence of glucose. Analysis of growth at pH 3 clearly shows that Pma1 function is generally intact in *ist2Δ* cells.

How can Ist2 influence the rate of H^+^ pumping under different environmental challenges? The association of Ist2 containing ER with Pma1 containing domains of the PM may create a specific cytosolic microcompartment with a small volume. In this microcompartment transport processes across the ER membrane and the buffering capacity of both membranes may become relevant to local pH and hence secondary active transport processes. In mammalian cells the primary ion gradients across the PM are established by the Na,K-ATPase and a direct coupling between Na,K-ATPase activity and amino acid transport has been described [50]. Besides Ist2-dependent association of ER with Pma1 containing domains of the PM, we observed that a certain area of MCP remained free of underlying cortical ER. These domains may function in processes like endo- and exocytosis, which depend on a free access to the PM from the cytosol and vice versa. Consistently, recent data showed that the cortical ER is dynamic but segregates from endocytosis initiation sites and the MCC [20], [24]. Both, the expression of human ANO1 in *ist2Δ* cells and the expression of truncated Ist2, lacking the first six TMDs, led to a partial rescue of the *ist2Δ* phenotype. This suggests that Ist2 has two different functions: One as channel and another one in the recruitment of cortical ER to the PM. Since ANO1 functions as a channel a flux of anions through Ist2 in either direction across the ER membrane is possible. Further insights into the subcellular distribution of chloride [51] and putative chloride gradients across yeast intracellular membranes are required to tackle the integration of ion transport processes across the ER membrane.

A flux of anions out of the cytosolic microcompartment between cortical ER and PM may reduce the electrogenic aspect of Pma1 function: Proton pumping leads to a strong hyperpolarization of the membrane. A removal of negative charges would enhance the ability of the Pma1 pump to transport protons against the force of the electric field. Secondary uptake of nutrients may depend on such a transient modulation of ion composition in the space between cortical ER and PM. More efficient nutrient uptake may assist the adaptation to growth under changing environments. Alternatively to an ion channel-based function, Ist2 may influence the lipid composition of the cortical ER or the PM. This hypothesis is raised by the recently described role of PM-localized ANO6 in asymmetric phospholipid distribution [11]. Changes of the lipid composition or protonation state at ER-PM contact sites could explain an influence of Ist2 on the localization and activity of Pma1. Decreased cytosolic pH was shown to result in release of the inositol transcription factor Opi1 from phosphatidic acid at ER-PM contact sites [52].

We found that invaginations of the PM, corresponding to the MCC, were often separated from or adjacent to the underlying cortical ER, marked by Ist2 and Sec63. This segregation between MCC and cortical ER has been observed previously [20], [24]. Interestingly, this distribution was maintained under conditions with changing amounts of cortical ER. The MCC contains a number of amino acid transporters including its defining member, the arginine permease Can1 [22], [23], [25]. Amino acid/H^+^ symporters of the MCC are separated from Pma1 in the PM [24]. This separation may help to locally couple specific ion transport processes in different domains of the PM. Impaired uptake of amino acids and other nutrients may explain the growth delay of *ist2Δ* cells during the adaptation to changing growth conditions.

In summary, we found that Ist2 recruits ER to the PM. Close association of ER and PM may contribute to specific metabolic functions of the PM.

## Materials and Methods

### Yeast strains, media and plasmids

All experiments were performed in BY4741/42 strain background [53]. The *IST2* gene was replaced by a natNT2 resistance gene in BY4742 using a PCR-based tagging method resulting in MSY399 [54]. Similarly, the genes *PMA1*, *SUR7* and *YOP1* were tagged by chromosomal integration of PCR amplified cassettes coding for either GFP or mCherry [54]. For constitutive overexpression of *IST2* we integrated BsiWI digested pMS623 into the *his3Δ1* locus of BY4742 resulting in MSY453. Isogenic WT (MSY474) and *IST2* deletion mutant (MSY483) were generated by integration of BsiWI digested pRS303 [55] into BY4742 and MSY399, respectively. Deletion of *IST2* (MSY399) was combined with *btn1Δ*, *btn2Δ*, *pmr1Δ*, *cnb1Δ*, *yck1Δ*, and *hrk1Δ* from the BY4741 deletion library by standard genetics [56]. As *pma1-007* hypomorph we used the *YGL007w* strain from the BY4741 deletion library. The preparations of synthetic HC media and yeast transformation were carried out as described [15]. The pH of YPD plates was adjusted with 50 mM 2-(N-morpholino)ethanesulfonic acid (MES) and KOH and HCl. Plasmid constructions are described in table S1.

### TIRF microscopy

Yeast cells expressing fluorescent protein tagged proteins were grown overnight at 25°C in HC media to mid log phase and immobilized on cover glasses with Concavanalin A (Sigma, Steinheim, Germany; lot# 080M7680V). Cover glasses were incubated with Concanavalin A for 1 min, dried for 1 min, and washed twice with water. 0.2 OD_600_ yeast culture was placed on coated cover glasses for 15 to 30 min, washed once with the respective media and mounted on glass slides with a thin layer of medium. For imaging an Olympus IX81 CellTirf microscope equipped with a 100×1.4 numerical aperture (NA) oil objective lens (Olympus) and an ImagEM (C9100-13) camera (Hamamatsu) driven by Olympus xcellence software was used. GFP and mCherry fluorescence were excited with a 50 mW 488 nm diode laser (Olympus) and a 100 mW 561 nm diode laser (Cobolt, Solna, Sweden), respectively. Emission light was filtered using a multiband filter set for the appropriate wavelengths (AHF, Tübingen, Germany). Bright field images were acquired using DIC optics. Image processing was performed using ImageJ (http://rsb.info.nih.gov/ij/).

### Confocal laser scanning microscopy and image analysis

Confocal imaging was performed with a Zeiss LSM 780 microscope using a 100×1.40 NA Plan-Apochromat oil objective lens (Zeiss) and pinhole settings of 1 airy unit. GFP fluorescence was excited with the 488 nm line of the argon laser and mCherry fluorescence was excited with a 561 nm diode-pump solid-state laser. Image processing and analysis was performed using ImageJ. For area measurements, the images were analyzed by performing the following protocol. First, a mean filter of the 3×3 pixel neighborhood (‘smooth’) was applied to reduce pixel outlier intensities and shot noise. Second, the images were background subtracted by using identical subtraction parameters (i.e. minus 30% of maximal pixel intensity values) for all images. Third, the areas of the different image channels were measured by intensity thresholding against a background set to intensity value zero. Regions-of-interest (ROI) were applied, representing the individual cell sizes, in order to measure areas and percentages of areas per whole cell size, respectively.

For colocalization analysis, the set of plugins from the Wright Cell Imaging Facility for ImageJ (www.uhnresearch.ca/wcif) was applied. In short, the background-subtracted two-channel images were analyzed for colocalization at ratio settings of 20% and thresholds of zero for both channels. Areas fulfilling these colozalisation criteria were then measured against the corresponding Sur7 areas in order to determine the degree of colocalization. Only Sur7 domains with a maximal diameter of 500 nm that were clearly separated from neighboring domains were analyzed. The degree of colocalization was grouped and labeled as full colocalization at 81–100% of signal overlap, 21–80% as adjacent and 0–20% as separated.

### Epifluorescence microscopy

Epifluorescence microscopy was performed with an Olympus xcellence IX81 microscope system using a 100×/1.45 NA Plan-Apochromat oil objective lens (Olympus) and a GFP/mCherry sbx ET filter set (AHF, Tübingen, Germany). As fluorescence light source the illumination system MT20 (Olympus) with a 150 W Xe arc burner was used. In order to colocalize Ist2 and ER proteins in the cortical ER, we acquired images of cell surfaces in focus. The Pearsons' coefficient was determined using the JACoP plugin [57] in ImageJ.

### Transmission Electron Microscopy

For immuno-EM yeast cells were grown to mid log phase in YPD medium at 30°C, concentrated by filtration and chemically fixed according to [58]. In detail, concentrated cells were mixed with double-concentrated fixative [4% (w/v) paraformaldehyde, 0.4% (v/v) glutaraldehyde in 0.1 M PHEM buffer (20 mM PIPES, 50 mM HEPES, pH 6.9, 20 mM EGTA, 4 mM MgCl_2_)] and incubated at room temperature for 30 min. Then, cells were pelleted, resuspended in standard fixative (2% (w/v) paraformaldehyde, 0.2% (v/v) glutaraldehyde in 0.1 M PHEM buffer) and incubated at 4°C overnight. Fixed cells were washed 3 times in PHEM buffer and incubated in 1% (w/v) periodic acid in PHEM buffer for 1 hour at 4°C and again washed 3 times. Cells were then embedded in 12% (w/v) gelatine in PHEM buffer, cut into small blocks, infused with 2.3 M sucrose in 0.1 M PHEM buffer at 4°C overnight, mounted on metal pins and frozen in liquid nitrogen. Ultrathin cryo-sections (75 nm) were cut using a cryo-ultramicrotome (Ultracut UCT with EM FCS, Leica) and a diamond knife (Diatome) at −110°C and placed on formvar-coated nickel grids. For immuno-labeling, sections were incubated with polyclonal rabbit Ist2 anti-serum [59] for 20 min, followed by incubation with protein A-10 nm gold (CMC, Utrecht) for 20 min. Sections were contrasted with 0.4% (w/v) uranyl acetate in 2 M methyl-cellulose for 10 min on ice and embedded in the same solution.

For Epon embedding, cells were grown as described above, rapidly filtered and vitrified using an EM HPM100 (Leica) high-pressure freezer. Fixed cells were further processed by freeze substitution in a Leica EM AFS, i.e. incubated in 0.5% glutaraldehyde, 1% H_2_O in acetone at −90°C for 70 hours, followed by incubation in 0.5% glutaraldehyde, 0.1% uranyl acetate, 5% H_2_O in acetone for 7 hours. Temperature was raised to −20°C (+5°C/hour), kept constant for 18 hours, and further raised to 4°C (+10°C/hour). Samples were washed 3 times with acetone, twice with propylene oxide and metal HPF planchets were removed. Samples were first infiltrated with 50% Epon resin in propylene oxide for 1 hour at room temperature, then with 100% Epon for an additional hour, and with fresh Epon resin overnight. After replacement with fresh resin, samples were incubated for 30 hours at 60°C. 70 nm ultrathin sections were cut with an ultramicrotome (Ultracut UCT, Leica) and contrasted with lead citrate.

All sections were examined with a Philips CM120 transmission electron microscope and micrographs were acquired with a CCD camera (Megaview III, Olympus Soft Imaging Systems). Image processing was performed using iTEM software (Olympus Soft Imaging Systems).

### Generation of yeast membranes and Western blotting

For fractionation, yeast cells were disrupted in lysis buffer (50 mM Hepes-KOH pH 7.6, 50 mM potassium acetate, 5 mM magnesium acetate, 1 mM EDTA, 1 mM DTT, 0.1 mM PMSF, Complete Protease Inhibitor (Roche)) and pelleted at 25000 g [59] [59]. Membrane fractions were resuspended in HU-buffer (8 M urea, 5% (w/v) SDS, 200 mM Tris–HCl, pH 6.8, 1 mM EDTA, 0.05% (w/v) bromphenol blue, 4% (v/v) β-mercaptoethanol), incubated for 10 min at 50°C and separated by 7.5% SDS–PAGE followed by Western blotting using Ist2- (1∶25000), Sec61- (1∶7500 [59], [60]), GFP- (1∶20,000, rabbit serum, gift from Dirk Görlich, Max-Planck-Institute for Biophysical Chemistry, Göttingen, Germany), Pma1 (1∶10.000, rabbit serum, provided by Ramón Serrano, Universidad Politécnica de Valencia, Valencia, Spain) or ANO1-specifc antibodies. The ANO1-specific antibodies were raised in a rabbit against KLKQQSPPDHEECVKRKQRYEVDY and CRYKDYREPPWSENK peptides. Detection and quantification of protein bands from Western blots was done using the LAS-1000 system (Fujifilm) followed by image processing using Adobe Photoshop and ImageJ software.

### pH measurements

Cytosolic pH was measured using pH-sensitive GFP (pHluorin, [34]) expressed under the control of the *TEF1* promoter from a *CEN* plasmid (pMS588). Fluorescence intensities at excitation wavelengths of 390 and 480 nm were measured at a constant emission wavelength of 520 nm in a FLUOstar Omega plate reader (BMG). Calibration of fluorescence with pH was carried out with 0.1% digitonin permeabilized cells in HC media buffered with 50 mM 3-(N-morpholino)propanesulfonic acid to 6.0, 6.5, 7.0, and 7.5.

## Supporting Information

Figure S1 **Log growth of** ***pTEF1-IST2*** **is similar to WT.** (A–B) *ist2Δ* cells were transformed with *HIS3* or *URA3 CEN* plasmids encoding *IST2* resulting in strains *ist2Δ+IST2 (CEN-HIS3)* and *ist2Δ+IST2 (CEN-URA3)*. (A) Growth of *ist2Δ+IST2 (CEN-HIS3)* and *ist2Δ+IST2 (CEN-URA3)* relative to WT (set to 100%) after 10 hours. Cultures were diluted to OD_600_ 0.05 from pre-cultures with an OD_600_ 1.0–2.0 (log), OD_600_ 2.0–2.5 (late log), OD_600_ 2.5–3.0 (transition), and OD_600_>3.0 (stationary). Error bars depict s.d., non-significant differences are indicated as n.s. (B) Membranes prepared from 5 OD_600_ of WT cells transformed with an empty plasmid (pRS303), and *ist2Δ+IST2 (CEN-HIS3)*, and *ist2Δ+IST2 (CEN-URA3)* were separated. Ist2 and Sec61 were detected with specific antibodies. (C) Stationary cultures (OD_600_>3.0) were diluted to 0.05 OD_600_ and grown at 25°C in HC complete medium for 30 hours. Growth curves are plotted with linear x-axis (time [h]) and logarithmic ordinate (OD_600_).
(TIF)

Figure S2 **Overexpression of Ist2 led to an increase of cortical ER.** (A) Quantification of the length of the PM with underlying cortical ER containing GFP-HDEL, Sec63-GFP or GFP-Ubc6. The ER proteins GFP-HDEL, Sec63-GFP, and GFP-Ubc6 were localized in *ist2Δ*, WT, and *pTEF1-IST2* cells using epifluorescence microscopy. The intensities of the peripheral signals of equatorial sections were quantified. Areas with more than 70% of the average peripheral signal intensity were classified as PM with an underlying cortical ER. For comparison the quantification of cortical ER by EM from figure 2H is shown as black bars. (B) Examples of *ist2Δ*, WT, *pTEF1-IST2* strains expressing GFP-Ubc6. Intensity profiles of peripheral staining as indicated by arrows in left panels are plotted in right panels. The percentage of PM with and without an underlying cortical ER is shown as cER and PM, respectively. (**C**) Representative z-stack CLS images of *ist2Δ* and *pTEF1-IST2* cells. All cells express the PM-marker Pma1-mCherry (in magenta) and coexpress either GFP-HDEL, Sec63-GFP, GFP-Ubc6 or GFP-Scs2 (all shown in green). The scale bar corresponds to 2 µm.
(TIF)

Figure S3 **Inheritance of cortical ER into the growing bud is independent of Ist2.** (A) Quantification of fluorescence ratios between daughter and mother cells in *ist2Δ* (white), WT (black) and *pTEF1-IST2* (grey) cells expressing both Yop1-mCherry and Sec63-GFP. Daughter cells were classified as small (S; <0.4 daughter perimeter/mother perimeter), medium (M; 0.4–0.6) and large (L; >0.6). (B–D) Representative z-stack CLS images (pinhole settings of 2 airy units) of *ist2Δ*, WT and *pTEF1-IST2* cells with small (B), medium (C) and large (D) daughters expressing both Yop1-mCherry (left panels; magenta in merge panels) and Sec63-GFP (middle panels; green in merge panels). (E) Immunoblot analysis of 5.0 OD_600_ membranes from *ist2Δ*, WT and *pTEF1-IST2* cells. Yop1-GFP (upper panel) and Sec61 (lower panel) were detected by GFP and Sec61 specific antibodies. Quantifications are shown as mean ± s.d. (n = 4).
(TIF)

Figure S4 **Membrane proteins localize in distinct domains of the cortical ER.** (A) GFP-Ist2 and mCherry-Ist2 rescue the growth phenotype of *ist2Δ.* WT (black), *ist2Δ* (grey) and *ist2Δ* cells with either genomically integrated *GFP-IST2* (green) or *mCherry-IST2* (red) were diluted to 0.05 OD_600_ from stationary pre-cultures in complete HC media and incubated at 25°C. The relative growth after 10 hours is shown with WT set to 100% ± s.d. (n = 4). (B) Table showing Pearson's coefficients of colocalization of indicated proteins tagged with GFP or mCherry. Mean values ± s.d. were quantified from indicated numbers of cells. Pairs of GFP or mCherry-tagged proteins were expressed in WT cells. (C–H) Surface views of WT cells expressing both dsRed-HDEL (C, E and G; magenta in merge and profiles) or Yop1-mCherry (D, F and H; magenta in merge and profiles) and GFP-HDEL (C and D; green in merge and profiles), Sec63-GFP (E and F; green in merge and profiles) or GFP-GFP-Ubc6 (G and H; green in merge and profiles) analyzed by epifluorescence microscopy. Intensity profiles of the indicated regions (white in merge panels) are shown in right panels. The scale bar corresponds to 2 µm.
(TIF)

Figure S5 **Sur7 is partially separated from cortical ER.** (A–D) Surface views of single cells imaged by CLS microscopy. (A) WT cells expressing Sur7-mCherry and GFP-Ist2. (B) WT cells expressing Sur7-mCherry and Sec63-GFP. (C) *ist2*Δ cells expressing Sur7-mCherry and Sec63-GFP. (D) *pTEF1-IST2* cells expressing Sur7-mCherry and Sec63-GFP. (E) Amount of cell surface covered with Sur7-mCherry (in magenta) and GFP-Ist2 or Sec63-GFP (in green) in the indicated strains calculated from the cells in A–D.
(TIF)

Figure S6 **Ist2 function is connected to pH homeostasis.** (A) Pma1 activation depends on glucose. Kinetic pH measurement of glucose-starved cells after addition of 2% galactose. Cells were starved for glucose for 1 hour in synthetic media. At the indicated time 2% galactose was added (marked with arrow). (B) Immunoblot analysis of 5.0 OD_600_ membranes from *ist2Δ*, WT, *pTEF1-IST2* and *pma1-007* cells. Pma1 (upper panel) and Sec61 (lower panel) were detected by Pma1 and Sec61 specific antibodies. Quantifications are shown as mean ± s.d. (n = 9). (C) Genetic interaction between *ist2Δ*, *btn1Δ* and *btn2Δ* mutants. Growth of five-fold serial dilutions of the indicated strains on plates with YPD and YDP+1 M NaCl. (D) Genetic interaction between *ist2Δ* and mutants lacking genes involved in Ca^2+^ homeostasis and signaling. Growth of serial dilutions of the indicated strains on plates with YPD and YDP+300 mM NaCl with an adjusted pH of 3.0 or 7.5. (E) Transformation of *ist2Δ* cells with plasmids encoding ANO1 did not result in the detection of ANO1 protein. Immunoblot analysis of 5 OD_600_ membranes from *ist2Δ* cells or *ist2Δ* cells transformed with an ANO1-encoding plasmid and lysate from HEK293 cells or HEK293 cells transfected with a GFP-ANO1-encoding plasmid. ANO1 protein and Sec61 were detected with ANO1-, GFP- and Sec61-specific antibodies. (F) Z-stack CLS images of *ist2Δ* transformed with a N-terminally mCherry-tagged truncated version of Ist2, lacking the N-terminal domain and TMDs 1–6 (amino acids 1–476 are deleted) in surface (left panel) and equatorial (middle panel) and phase contrast (PC; right panel) views are shown.
(TIF)

Table S1 **Plasmids used in this study.**
(DOC)
