# Supplementary figures and images for: Yeast Ist2 Recruits the Endoplasmic Reticulum to the Plasma Membrane and Creates a Ribosome-Free Membrane Microcompartment
Source: PLoS One. 2012 Jul 9;7(7):e39703. doi: 10.1371/journal.pone.0039703 (PMC3392263; doi:10.1371/journal.pone.0039703)

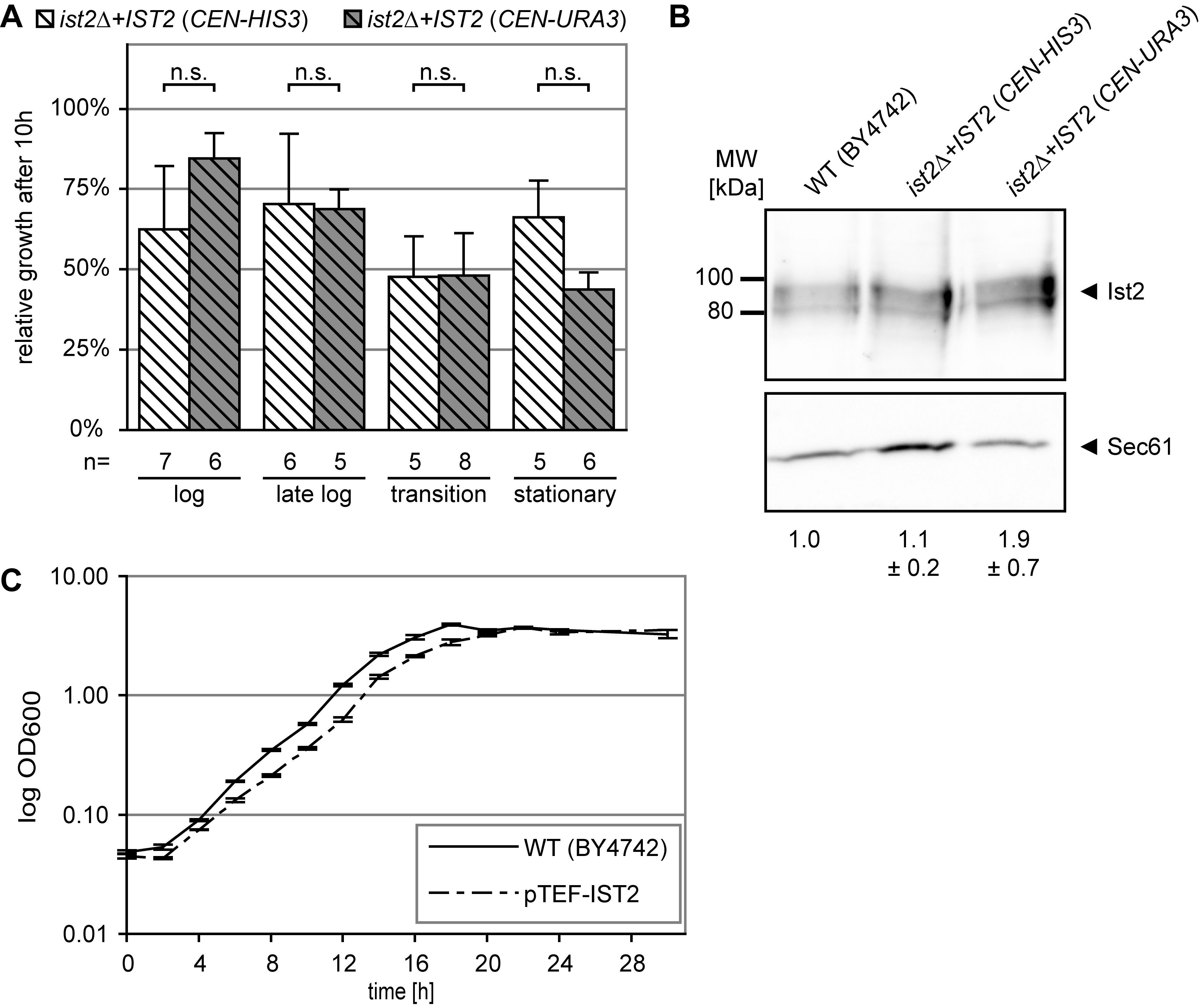

Supplement: Figure S1 — Log growth of pTEF1-IST2 is similar to WT. (A–B) ist2Δ cells were transformed with HIS3 or URA3 CEN plasmids encoding IST2 resulting in strains ist2Δ+IST2 (CEN-HIS3) and ist2Δ+IST2 (CEN-URA3). (A) Growth of ist2Δ+IST2 (CEN-HIS3) and ist2Δ+IST2 (CEN-URA3) relative to WT (set to 100%) after 10 hours. Cultures were diluted to OD600 0.05 from pre-cultures with an OD600 1.0–2.0 (log), OD600 2.0–2.5 (late log), OD600 2.5–3.0 (transition), and OD600>3.0 (stationary). Error bars depict s.d., non-significant differences are indicated as n.s. (B) Membranes prepared from 5 OD600 of WT cells transformed with an empty plasmid (pRS303), and ist2Δ+IST2 (CEN-HIS3), and ist2Δ+IST2 (CEN-URA3) were separated. Ist2 and Sec61 were detected with specific antibodies. (C) Stationary cultures (OD600>3.0) were diluted to 0.05 OD600 and grown at 25°C in HC complete medium for 30 hours. Growth curves are plotted with linear x-axis (time [h]) and logarithmic ordinate (OD600). (TIF) [file pone.0039703.s001.tif]

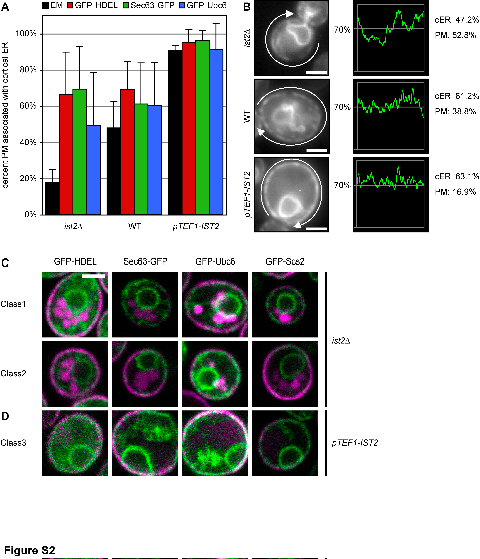

Supplement: Figure S2 — Overexpression of Ist2 led to an increase of cortical ER. (A) Quantification of the length of the PM with underlying cortical ER containing GFP-HDEL, Sec63-GFP or GFP-Ubc6. The ER proteins GFP-HDEL, Sec63-GFP, and GFP-Ubc6 were localized in ist2Δ, WT, and pTEF1-IST2 cells using epifluorescence microscopy. The intensities of the peripheral signals of equatorial sections were quantified. Areas with more than 70% of the average peripheral signal intensity were classified as PM with an underlying cortical ER. For comparison the quantification of cortical ER by EM from figure 2H is shown as black bars. (B) Examples of ist2Δ, WT, pTEF1-IST2 strains expressing GFP-Ubc6. Intensity profiles of peripheral staining as indicated by arrows in left panels are plotted in right panels. The percentage of PM with and without an underlying cortical ER is shown as cER and PM, respectively. (C) Representative z-stack CLS images of ist2Δ and pTEF1-IST2 cells. All cells express the PM-marker Pma1-mCherry (in magenta) and coexpress either GFP-HDEL, Sec63-GFP, GFP-Ubc6 or GFP-Scs2 (all shown in green). The scale bar corresponds to 2 µm. (TIF) [file pone.0039703.s002.tif]

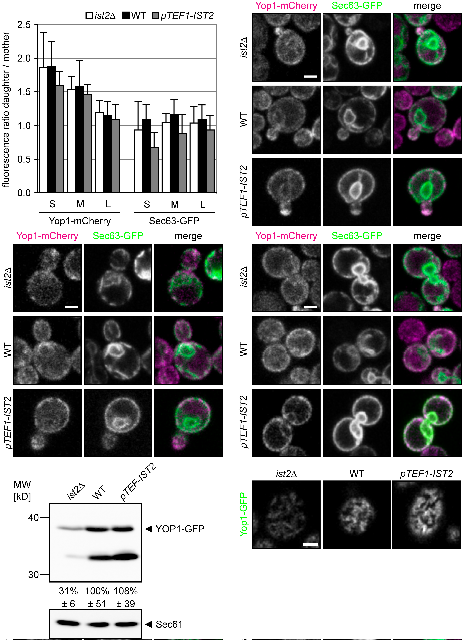

Supplement: Figure S3 — Inheritance of cortical ER into the growing bud is independent of Ist2. (A) Quantification of fluorescence ratios between daughter and mother cells in ist2Δ (white), WT (black) and pTEF1-IST2 (grey) cells expressing both Yop1-mCherry and Sec63-GFP. Daughter cells were classified as small (S; <0.4 daughter perimeter/mother perimeter), medium (M; 0.4–0.6) and large (L; >0.6). (B–D) Representative z-stack CLS images (pinhole settings of 2 airy units) of ist2Δ, WT and pTEF1-IST2 cells with small (B), medium (C) and large (D) daughters expressing both Yop1-mCherry (left panels; magenta in merge panels) and Sec63-GFP (middle panels; green in merge panels). (E) Immunoblot analysis of 5.0 OD600 membranes from ist2Δ, WT and pTEF1-IST2 cells. Yop1-GFP (upper panel) and Sec61 (lower panel) were detected by GFP and Sec61 specific antibodies. Quantifications are shown as mean ± s.d. (n = 4). (TIF) [file pone.0039703.s003.tif]

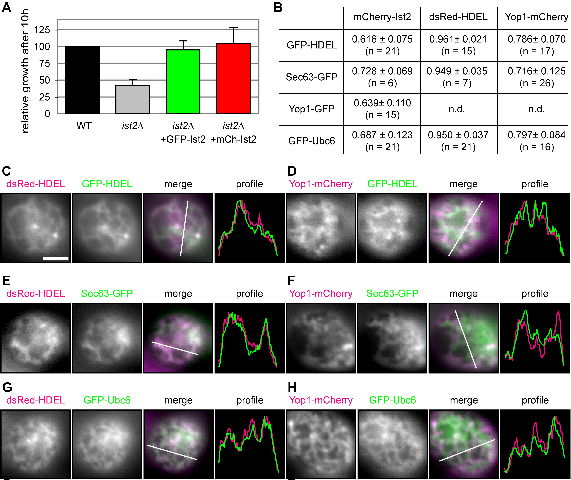

Supplement: Figure S4 — Membrane proteins localize in distinct domains of the cortical ER. (A) GFP-Ist2 and mCherry-Ist2 rescue the growth phenotype of ist2Δ. WT (black), ist2Δ (grey) and ist2Δ cells with either genomically integrated GFP-IST2 (green) or mCherry-IST2 (red) were diluted to 0.05 OD600 from stationary pre-cultures in complete HC media and incubated at 25°C. The relative growth after 10 hours is shown with WT set to 100% ± s.d. (n = 4). (B) Table showing Pearson's coefficients of colocalization of indicated proteins tagged with GFP or mCherry. Mean values ± s.d. were quantified from indicated numbers of cells. Pairs of GFP or mCherry-tagged proteins were expressed in WT cells. (C–H) Surface views of WT cells expressing both dsRed-HDEL (C, E and G; magenta in merge and profiles) or Yop1-mCherry (D, F and H; magenta in merge and profiles) and GFP-HDEL (C and D; green in merge and profiles), Sec63-GFP (E and F; green in merge and profiles) or GFP-GFP-Ubc6 (G and H; green in merge and profiles) analyzed by epifluorescence microscopy. Intensity profiles of the indicated regions (white in merge panels) are shown in right panels. The scale bar corresponds to 2 µm. (TIF) [file pone.0039703.s004.tif]

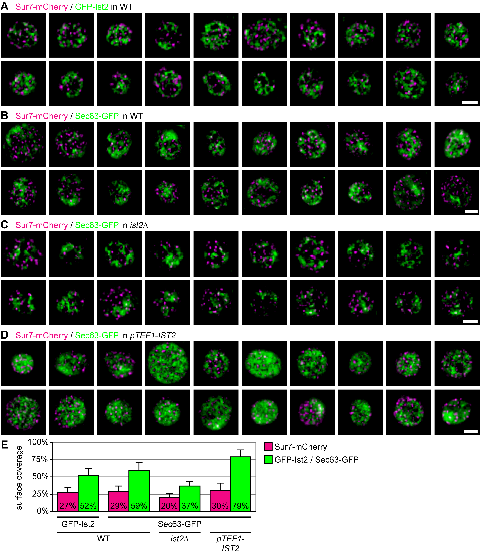

Supplement: Figure S5 — Sur7 is partially separated from cortical ER. (A–D) Surface views of single cells imaged by CLS microscopy. (A) WT cells expressing Sur7-mCherry and GFP-Ist2. (B) WT cells expressing Sur7-mCherry and Sec63-GFP. (C) ist2Δ cells expressing Sur7-mCherry and Sec63-GFP. (D) pTEF1-IST2 cells expressing Sur7-mCherry and Sec63-GFP. (E) Amount of cell surface covered with Sur7-mCherry (in magenta) and GFP-Ist2 or Sec63-GFP (in green) in the indicated strains calculated from the cells in A–D. (TIF) [file pone.0039703.s005.tif]

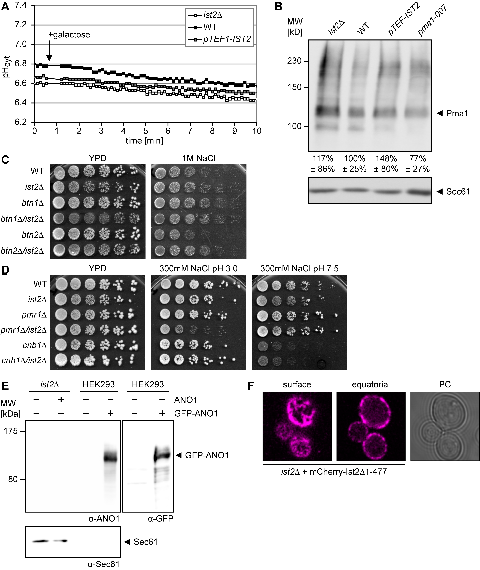

Supplement: Figure S6 — Ist2 function is connected to pH homeostasis. (A) Pma1 activation depends on glucose. Kinetic pH measurement of glucose-starved cells after addition of 2% galactose. Cells were starved for glucose for 1 hour in synthetic media. At the indicated time 2% galactose was added (marked with arrow). (B) Immunoblot analysis of 5.0 OD600 membranes from ist2Δ, WT, pTEF1-IST2 and pma1-007 cells. Pma1 (upper panel) and Sec61 (lower panel) were detected by Pma1 and Sec61 specific antibodies. Quantifications are shown as mean ± s.d. (n = 9). (C) Genetic interaction between ist2Δ, btn1Δ and btn2Δ mutants. Growth of five-fold serial dilutions of the indicated strains on plates with YPD and YDP+1 M NaCl. (D) Genetic interaction between ist2Δ and mutants lacking genes involved in Ca2+ homeostasis and signaling. Growth of serial dilutions of the indicated strains on plates with YPD and YDP+300 mM NaCl with an adjusted pH of 3.0 or 7.5. (E) Transformation of ist2Δ cells with plasmids encoding ANO1 did not result in the detection of ANO1 protein. Immunoblot analysis of 5 OD600 membranes from ist2Δ cells or ist2Δ cells transformed with an ANO1-encoding plasmid and lysate from HEK293 cells or HEK293 cells transfected with a GFP-ANO1-encoding plasmid. ANO1 protein and Sec61 were detected with ANO1-, GFP- and Sec61-specific antibodies. (F) Z-stack CLS images of ist2Δ transformed with a N-terminally mCherry-tagged truncated version of Ist2, lacking the N-terminal domain and TMDs 1–6 (amino acids 1–476 are deleted) in surface (left panel) and equatorial (middle panel) and phase contrast (PC; right panel) views are shown. (TIF) [file pone.0039703.s006.tif]
