# Supplementary material for: Yeast Ist2 Recruits the Endoplasmic Reticulum to the Plasma Membrane and Creates a Ribosome-Free Membrane Microcompartment
Source: PLoS One. 2012 Jul 9;7(7):e39703. doi: 10.1371/journal.pone.0039703 (PMC3392263; doi:10.1371/journal.pone.0039703)
Supplement: Table S1 — Plasmids used in this study. (DOC) [file pone.0039703.s007.doc]

Table S1: Plasmids used in this study

| plasmid | insert (restriction sites used) | 5’UTR (restriction sites used) | 3’UTR (restriction sites used) | backbone |
| --- | --- | --- | --- | --- |
| e | GFP-HDEL | *MET25*^[2[^ |  |  |
| e | *SEC63*-GFP |  |  |  |
| pCJ22 | *IST2* (BamHI/ XhoI) | *IST2* | *IST2* | pRS316^[1]^ |
| pMS555 | *IST2* (BamHI/ NheI) | *IST2* (SacI/ BamHI) | *IST2* (NheI/ XhoI) | pRS313^[1]^ |
| pMS588 | pHluorin^[4]^ | *TEF1*^[2]^ |  | p415^[2]^ |
| pMS601 | *IST2* (BamHI/ NheI) | *IST2* (500 bp upstream ORF; SacI/ BamHI) | *IST2* (500 bp downstream ORF; NheI/ XhoI) | pRS303^[1],a^ |
| pMS602 | yeGFP-*IST2* (XbaI/ NheI) | *IST2* (SacI/ XbaI) | *IST2* (NheI/ XhoI) | pRS303^[1],a^ |
| pMS615 | ANO1 (BamHI/ NheI) | *IST2* (SacI/ BamHI) | *IST2* (NheI/ XhoI) | pRS303^[1],a^ |
| pMS623 | *IST2* (BamHI/ NheI) | *TEF1*^d^ (SacI/ BamHI) | *IST2* (NheI/ XhoI) | pRS303^[1],a^ |
| pMS624 | ANO1+CSS^b^ (BamHI/ NheI) | *IST2* (SacI/ BamHI) | *IST2* (NheI/ XhoI) | pRS303^[1],a^ |
| pMS648 | GFP-*SCS2* | *ACT1*^[2]^ | *ACT1*^[2]^ | pRS316 |
| pMS656 | GFP-*UBC6* | *MET25*^[2]^ |  | p415^[2]^ |
| pMS666 | mCherry-*IST2* (XmaI/ NheI) | *IST2* (SacI/ XmaI) | *IST2* (NheI/ XhoI) | pRS303^[1],a^ |
| pMS701 | mCherry(XmaI/ BamHI)*-IST2*∆1-477(BamHI/ NheI) | *IST2* (SacI/ XmaI) | *IST2* (NheI/ XhoI) | pRS303^[1],a^ |

^a^ pRS303 plasmids except pMS666 were linearized by BsiWI restriction for integration into *his3∆1* locus. pMS666 was linearized by ClaI restriction.

^b^  The CSS contains the last 207 bp of the *IST2* ORF including the stop codon.

**References**

1. Sikorski RS, Hieter P (1989) A system of shuttle vectors and yeast host strains designed for efficient manipulation of DNA in Saccharomyces cerevisiae. Genetics 122: 19-27.

2. Mumberg D, Muller R, Funk M (1995) Yeast vectors for the controlled expression of heterologous proteins in different genetic backgrounds. Gene 156: 119-122.

3. Prinz WA, Grzyb L, Veenhuis M, Kahana JA, Silver PA, et al. (2000) Mutants affecting the structure of the cortical endoplasmic reticulum in Saccharomyces cerevisiae. J Cell Biol 150: 461-474.

4. Miesenbock G, De Angelis DA, Rothman JE (1998) Visualizing secretion and synaptic transmission with pH-sensitive green fluorescent proteins. Nature 394: 192-195.
